# Ethacrynic acid decreases expression of proinflammatory intestinal wall cytokines and ameliorates gastrointestinal stasis in murine postoperative ileus

**DOI:** 10.6061/clinics/2018/e332

**Published:** 2018-10-10

**Authors:** Tomoyuki Harada, Mitchell Fink, Ruy J Cruz

**Affiliations:** I Department of Critical Care and Emergency Medicine, Tokyo Woman's Medical University, Tokyo, Japan; II Department of Critical Care and Emergency Medicine, Dokkyo Medical University Koshigaya Hospital, Saitama, Japan; III Department of Critical Care Medicine, University of Pittsburgh School of Medicine, PA, USA; IV Department of Surgery, University of Pittsburgh School of Medicine, PA, USA; V Intestinal Rehabilitation and Transplant Center, Starzl Transplantation Institute, University of Pittsburgh School of Medicine, PA, USA

**Keywords:** Ileus, Gastrointestinal Motility, Ethacrynic Acid, Small Intestine, Inflammation Mediators

## Abstract

**OBJECTIVES:**

Several compounds characterized by an olefin linkage conjugated to a carbonyl group have anti-inflammatory properties. The diuretic ethacrynic acid (EA) is a compound of this type. Herein, we tested the hypothesis that ethacrynic acid can modulate the development of ileus after bowel manipulation.

**METHODS:**

Groups (n=9) of male C57Bl/6 mice underwent surgical manipulation of the small intestine using a pair of cotton-tipped applicators (MAN). Control animals (CONT) did not undergo any surgical intervention or receive treatment. MAN mice were pre- and post-treated with four intraperitoneal doses of phosphate buffered saline (PBS), EA1 (1mg/kg per dose), or EA10 (10mg/kg per dose). Gastrointestinal transit of non-absorbable FITC-labeled dextran was assessed by gavaging the mice with the tracer 24h after operation and assessing FD70 concentration 120 min later in the bowel contents from the stomach, 10 equally long segments of small intestine, cecum, and two equally long segments of colon. The geometric center for the tracer was calculated for each animal. Expression of interleukin-6 (IL-6) and inducible nitric oxide synthase (iNOS) transcripts in the ileal muscularis propria was assessed using semiquantitative reverse transcriptase-polymerase chain reaction.

**RESULTS:**

In control animals, the mean (±SE) geometric center for the transit marker was 9.89±0.47, whereas it was 4.59±0.59 for PBS-treated animals (*p*<0.05 *vs* CONT). The geometric center for pre- post treatment with low (1mg/kg) and high (10mg/kg) doses of ethacrynic acid were 7.23±0.97 and 5.15±0.57, respectively. Compared to PBS, treatment with ethacrynic acid (1mg/kg) significantly decreased manipulation-induced IL-6 and iNOS mRNA expression in the wall of the small bowel.

**CONCLUSIONS:**

Pre- and post-treatment with ethacrynic acid ameliorates ileus and modulates inflammation in the gut wall induced by bowel manipulation.

## INTRODUCTION

Manipulation of the bowel during abdominal surgery inevitably leads to a period of impairment of gastrointestinal (GI) tract peristalsis. Although ileus is generally accepted as a normal response to abdominal surgery, it is a factor that contributes to discomfort, abdominal distention, respiratory compromise, nausea, and emesis in the post-operative period 1-3. Post-operative ileus (POI) also substantially burdens healthcare institutions financially and resource-wise, estimated to cost approximately US$ 1.5 billion annually 4,5 POI is associated with an increased expression of proinflammatory cytokines and chemokines in the gut wall, as well as infiltration of polymorphonuclear leukocytes and macrophages into the muscularis propria 6,7. Several pharmacological interventions have been tested to minimize the postoperative inflammatory process within the intestinal wall and reduce POI 8-11.

Several compounds characterized by an olefin linkage conjugated to a carbonyl group have been used as anti-inflammatory agents. The diuretic ethacrynic acid (EA) is a compound of this type. Despite its potential anti-inflammatory properties, few studies have examined the possible benefits of EA on the modulation of the inflammatory process 12-14. A recent study by Han et al. showed that EA is a potent inhibitor of lipopolysaccharide-induced nuclear factor-kappa B (NF-kB) activation and inducible nitric oxide synthase (iNOS) expression in RAW 264.7 murine macrophage-like cells 12. The aim of this study was to test the hypothesis that EA can attenuate inflammation and ameliorate ileus after small bowel manipulation.

## MATERIALS AND METHODS

### Animals and treatment groups

**Figure 1:**
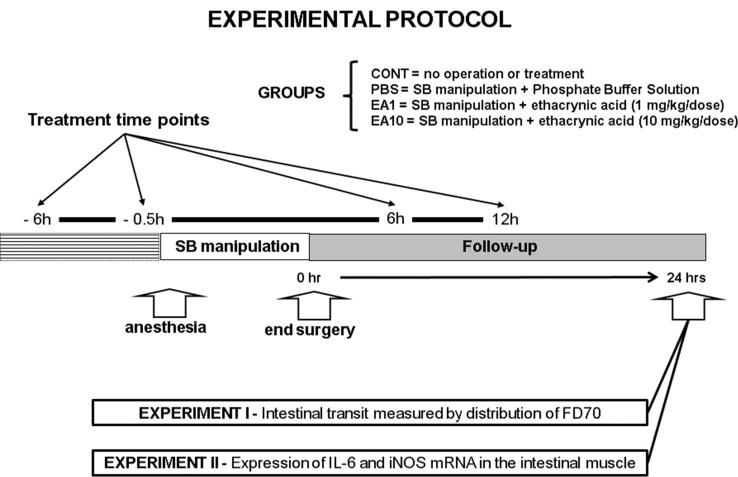
Schematic presentation of the experimental protocol. Mice were submitted to laparotomy and standardized small intestine manipulation. The animals were divided in four groups: CONT, no operation, no treatment; PBS, manipulation plus treatment with phosphate buffered saline solution; EA1, manipulation plus treatment with EA (1 mg/kg body weight per dose); and EA10, manipulation plus treatment with EA (10 mg/kg per dose,). All solutions were injected intraperitoneally at the same time points (-6h, –0.5h, +6h, and +12h) relative to the time of surgery. In the first experiment, intestinal transit was measured 24h after surgery by evaluating the distribution of orally fed FD70 (n=9 per condition). The second experiment, was designed to estimate the expressions of interleukin-6 (IL-6) and iNOS mRNA in the intestinal smooth muscle using semi-quantitative RT-PCR (n=8 per condition).

Male C57Bl/6 mice weighing 20–25 g were used in this study, which was approved by the University of Pittsburgh's Institutional Animal Use and Care Committee. The mice were anesthetized with intramuscular pentobarbital sodium (90 mg/kg). The small intestine was exteriorized through a midline abdominal incision and then manipulated in a standardized fashion with a pair of moist cotton applicators. After manipulation, the intestine was returned to its normal anatomic position within the peritoneal cavity and the incision was closed using a double-layer running suture. In the first experiment, we studied four groups of mice: CONT, no operation, no treatment (n=9); PBS, manipulation plus treatment with phosphate buffered saline (PBS, n=9) solution; EA1, manipulation plus treatment with EA (1 mg/kg body weight per dose, n=9) dissolved in PBS solution; and EA10, manipulation plus treatment with EA (10 mg/kg per dose, n=9). All solutions were injected intraperitoneally (i.p.) at the same time points (-6h, –0.5h, +6h, and +12h) relative to the time of surgery. The volume of solution injected intraperitoneally was identical in all treatment groups. In the second experiment, which was designed to assess semi-quantitative image results for reverse transcription polymerase chain reaction (RT-PCR), only three groups were studied: CONT, PBS, and EA1 (n=8, per condition) (Figure 1).

### Intestinal transit

Intestinal transit was measured 24h after surgery by evaluating the intestinal distribution of orally fed FD70, as previously described 8,9. After gavaging the mice for 120 min with 200 μl of FD70 dissolved in distilled water (2.5 mg/mL), the animals were euthanized with a halothane overdose. The entire GI tract from stomach to distal colon was excised and divided into 14 segments: the stomach, small intestine (divided into 10 segments of equal length), cecum, and colon (two segments of equal length). The luminal content of each segment was collected into a small tube and suspended in 1 mL of distilled water. The samples were mixed vigorously and then clarified with centrifugation (14,000*g* × 15min). The supernatants were collected and fluorometrically assayed for FD70 concentration using an excitation wavelength of 492nm (slit width, 2.5nm) and an emission wavelength of 515 nm (slit width, 10nm). The transit of FD70 along the GI tract was summarized by calculating the geometric center (GC) for the distribution: Σ(fraction of total recovered FD70 fluorescence in the *i*^th^ segment × *i*) 3.

### RT-PCR

The expressions of interleukin-6 (IL-6) and iNOS mRNA in the intestinal smooth muscle was estimated using semi-quantitative RT-PCR. The same procedure was used previously by our group to extract the RNA from the muscularis propria 8,9. In summary, segments of ileum were harvested 24 hours after manipulation or after the sham procedure. The small intestine was opened along the antimesenteric border and the mucosa was scraped away using a glass microscope slide. Total RNA was extracted from the muscularis propria with chloroform and TRI Reagent (Molecular Research Center, Cincinnati, OH) exactly as directed by the manufacturer. Total RNA was treated with DNAFree (Ambion, Houston, TX) as instructed by the manufacturer by using 10 units of DNase 1/10 lg RNA. Two lg of total RNA was reverse transcribed in a 40 lL reaction volume containing 0.5 lg of oligo(dT)15 (Promega, Madison, WI), 1 mmol/L of each deoxyribonucleoside triphosphate (dNTP), 15 U avian myeloblastosis virus reverse transcriptase (RT) (Promega), and 1 U/lL of recombinant RNasin ribonuclease inhibitor (Promega) in 5 mmol/L MgCl2, 10 mmol/L TRIS HCl, 50 mmol/L KCL, 0.1% Triton X-100, pH 8.0. The reaction mixtures were preincubated at 21°C for 10 minutes before DNA synthesis. The RT reactions were carried out for 50 minutes at 42°C and were heated to 95°C for five minutes to terminate the reaction. Reaction mixtures (50 lL) for polymerase chain reaction (PCR) were assembled with the use of 5 lL of complementary DNA template, 10 units AdvanTaq Plus DNA Polymerase (Clontech, Palo Alto, CA), 200 mmol/L of each dNTP, 1.5 mmol/L MgCl2, and 1.0 mmol/L of each primer in 1 3 AdvanTaq Plus PCR buffer. PCR reactions were performed using a GeneAmp Model 9700 thermocycler (Perkin Elmer, Norwalk, CT). Amplification was initiated with five minutes of denaturation at 94°C. The PCR conditions were denaturation at 94°C for 45 seconds, annealing at 61°C for 45 seconds, and polymerization at 72°C for 45 seconds. To ensure amplification was in the linear range, we empirically identified the optimal number of cycles. After the last amplification cycle, the samples were incubated at 72°C for 10 minutes and then held at 4°C. The 5# and 3# primers for IL-6 were CTG GTG ACA ACC ACG GCC TCC CCT and ATG CTT AGG CAT AAC GCA CTA GGT, respectively; the expected product length was 600 bp. The 5# and 3# primers for iNOS were CAC CAC AAG GCC ACA TCG GAT T and CCG ACC TGA TGT TGC CAT TGT T, respectively; the expected product length was 426 bp. 18S ribosomal RNA was amplified to verify equal loading. For this reaction, the 5# and 3# primers were CCC GGG GAG GTA GTG ACG AAA AAT and CGC CCG CTC CCA AGA TCC AAC TAC, respectively; the expected product length was 200 bp. Ten microliters of each PCR reaction was electrophoresed on a 2% agarose gel, scanned in NucleoVision imaging workstation (NucleoTech, San Mateo, CA), and quantified with the use of GelExpert release 3.5.

### Statistical analysis

Results are presented as means ±SE. Intestinal transit was analyzed by analysis of variance followed by Fisher's least significant difference test. The specific statistical approach used is indicated in the legend for the relevant figure. *P* values <0.05 were considered significant. Data obtained from RT-PCR were not analyzed statistically in view of the semiquantitative nature of the assay employed.

## RESULTS

### Gastrointestinal transit

**Figure 2:**
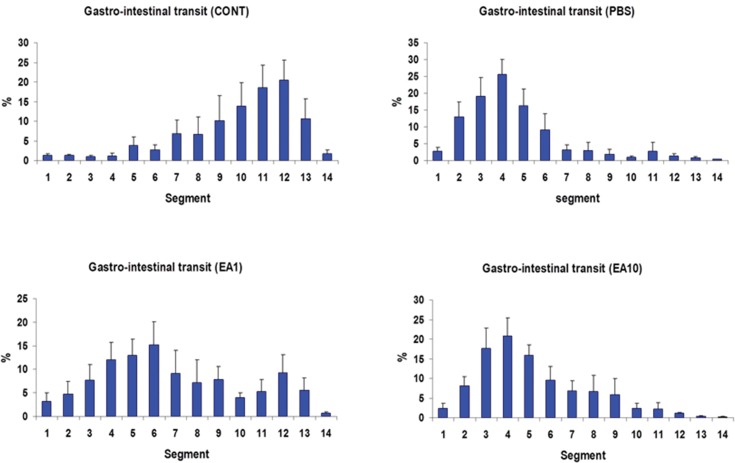
Distribution along the gastrointestinal tract of orally-administered FD70 in the CONT group and in animals subjected to bowel manipulation. Mice in the CONT group were not subjected to anesthesia and surgery and did not receive any treatment (n=9). Mice in the PBS group (n=9) were subjected to manipulation and treated with phosphate buffered saline solution. Mice in the EA1 group (n=9) were subjected to manipulation and treated with four 1 mg/kg doses of ethacrynic acid (EA). Mice in the EA10 group (n=9) were subjected to manipulation and treated with four 10 mg/kg doses of EA. All solutions were injected intraperitoneally at the same time points (-6h, –0.5h, +6h and +12h) relative to the time of surgery. Segment 1 is the stomach; segments 2-11 are equal lengths of small intestine from the duodenum to the ileocecal junction; segment 12 is the cecum; segment 13 is the proximal colon; and segment 14 is the distal colon. Results are presented as mean ±SE.

**Figure 3:**
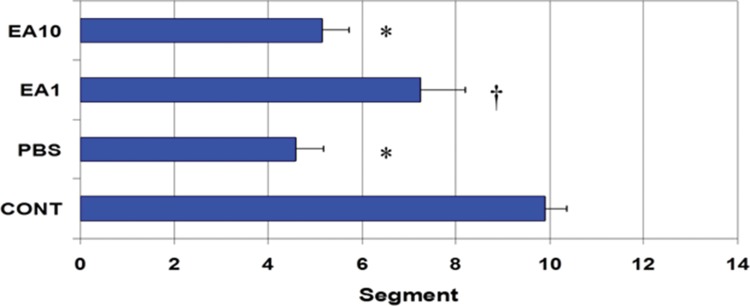
Mean ± SE of the geometric center (GC) for each treatment group shown in Figure 1. Higher numbers indicate better progression of the FD70 tracer into the distal gastrointestinal tract. *indicates *p*<0.05 *vs.* CONT and † indicates *p*<0.05 *vs.* PBS.

In the control group (*i.e.*, completely normal mice that were not subjected to anesthesia or laparotomy), the enterically-administered fluorescent tracer was rapidly transported in an aboral direction within the intestine such that the peak signal 90 min after administration was in the distal ileum and colon (segments 10 to 12; Figure 2A). In contrast, in the PBS group (*i.e.*, mice subjected to gut manipulation but treated only with PBS solution), the distribution of the fluorescent tracer was shifted toward the more proximal segments of the GI tract (segments 3 to 5, Figure 2B). Pre- post-treatment of mice with four doses of 1 mg/kg body weight per dose of ethacrynic acid (EA1) ameliorated the derangement in intestinal motility induced by surgical manipulation (Figures 2C). Increasing the doses of EA to 10mg/kg was not associated with any additional improvement in gut motility (Figure 2D). In control animals, the mean (±SE) GC for the transit marker was 9.89±0.47, whereas for PBS-treated animals it was 4.59±0.59 (*p*<0.05) (Figure 3). The GC for pre- post-treatment with low (1mg/kg) and high (10mg/kg) doses of EA were 7.23±0.97 and 5.15±0.57, respectively (Figure 3). The lower dose of EP (1mg/kg) tended to be more efficacious than the higher dose in increasing GI motility (Figures 2C, 2D, and 3).

### Expression of IL-6 and iNOS transcripts

**Figure 4:**
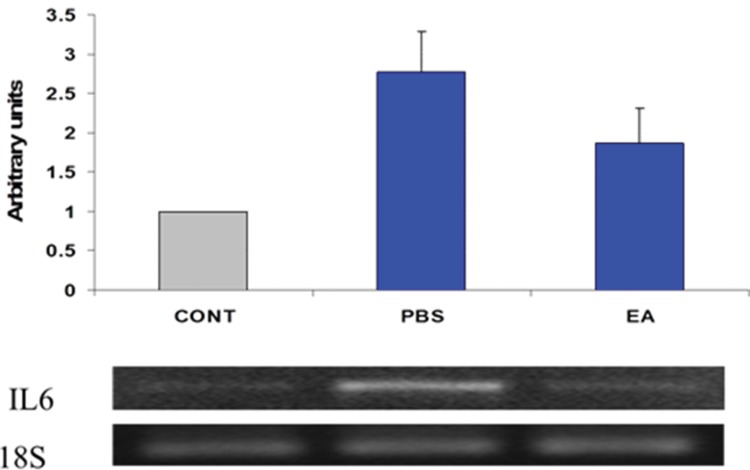
Expression of interleukin-6 (IL-6) mRNA in intestinal smooth muscle. Results were obtained using semi-quantitative RT-PCR. Bands were scanned in a NucleoVision imaging workstation (NucleoTech, San Mateo, CA) and quantified using GelExpert release 3.5. Data in bar graphs are mean ± SE (n=8 per condition).

**Figure 5:**
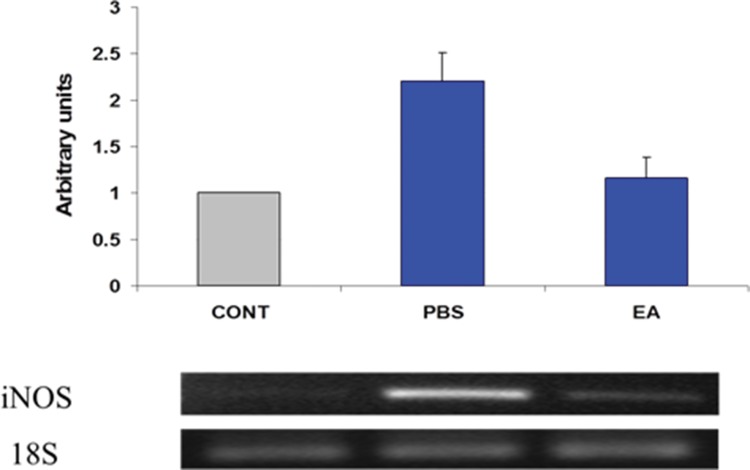
Expression of inducible nitric oxide synthase (iNOS) mRNA in intestinal smooth muscle. Results were obtained using semi-quantitative RT-PCR. Bands were scanned in a NucleoVision imaging workstation (NucleoTech, San Mateo, CA) and quantified using GelExpert release 3.5. Data in bar graphs are mean ±SE (n=8 per condition).

Estimates regarding changes in the expression of several pro-inflammatory gene products in the muscularis were determined using semi-quantitative RT-PCR. Twenty-four hours after surgery, gut manipulation was associated with increased expression of IL-6 and iNOS mRNA in the muscularis propria of the ileum (Figures 4 and 5, respectively). Pre- post-treatment of mice with four doses of EA (1 mg/kg body weight per dose) decreased the steady-state levels of transcripts for both pro-inflammatory genes.

## DISCUSSION

In this study, we observed that small bowel manipulation is associated with a marked increase in expression of IL-6 and iNOS transcripts in the ileal muscularis propria, assessed using semiquantitative RT-PCR. Treatment with 1mg/kg EA decreased the steady-state levels of transcripts for both pro-inflammatory genes. We also demonstrated that pre- and post-treatment with EA improves intestinal transit 24h after small bowel manipulation.

The development of POI is multifactorial, with an intricate interaction between inflammatory, hormonal, neurogenic, and humoral components. Several studies have shown that inflammation plays a critical role in the development of POI 8,10,11. In a recent review, Vather et al. 2 postulated that the mechanisms by which bowel wall inflammation causes dysmotility are threefold. First, several molecules involved in the inflammatory process are potent muscle relaxants and therefore have a direct impact on contractility. Secondly, intestinal wall edema (caused mainly by intraoperative fluid replacement and local trauma) is believed to mechanically impair the efficacy of myotonic contraction. Finally, changes in splanchnic perfusion during anesthesia and abdominal surgery (*i.e.* ischemia/reperfusion injury) may play a role in an ileus by exacerbating the inflammatory response.

In 1969, Oronsky et al. 14 reported one of the first studies assessing the potential anti-inflammatory effects of EA. The authors examined the effects of different sulfhydryl binding compounds (including EA) on the inflammatory response in rats during the first 24 hours after implanting a pellet in the subcutaneous tissue. Recent studies have shown that the anti-inflammatory effects of EA are attributed to the inhibition of multiple steps in the NF-kB-signaling pathway, as well as modulating leukotriene formation 12,13,15,16. The data presented herein showed a significant decrease in the steady state levels of IL-6 and iNOS mRNA in EA-treated mice compared with PBS-treated mice, a finding consistent with the view that treatment with this agent downregulated the inflammatory response in the smooth muscle layers of the bowel after surgical manipulation. Although it is impossible to be certain that the anti-inflammatory effects of EA were solely responsible for the salutary effects of this agent with respect to gut motility, this explanation seems to be highly plausible.

Two other pharmacological properties of EA could have also contributed to the attenuation of the inflammatory process on the intestinal wall and ameliorated POI. First, the diuretic effect of EA might have decreased post-operative intestinal edema with significant improvement in myotonic contraction. Secondly, inhibition of gluthadione S-tranferase by EA might have minimized splanchnic ischemia/reperfusion injury and inflammation 17. Therefore, we can speculate that the salutary effects of EA on the minimization of POI can be multifactorial, not just related to a decrease of expression of inflammatory cytokines within the intestinal wall. However, further mechanistic and pharmacological studies are necessary to confirm this hypothesis.

As mentioned previously, starting a low dose of EA (1 mg/kg) before the surgical procedure and continuing for 12 hours afterwards significantly improved small bowel transit in a murine model of POI, with a significant shift of the GC to the right when compared to PBS-treated animals (7.23±0.97 *vs.* 4.59±0.59, *p*<0.05). In the clinical setting, the recommended intravenous dose of EA ranges from 0.5 to 1 mg/kg, which matches the low dose used in this study. Unfortunately, experimental and/or clinical studies evaluating the dosing effect of EA on inflammatory modulation are lacking. Interestingly, a ten-fold increase in EA dose abrogated the benefits of the post-operative intestinal motility observed in animals treated with a lower dose. One of the possible explanations for this finding is that higher doses of EA (10 mg/kg) could have led to a substantial perioperative saliuresis and kaliuresis, with significant electrolyte imbalance and further perpetuation of POI. However, additional dose-response studies are necessary to validate these findings.

There are limitations in our experimental model. First, we used standardized pre- and post- operative doses, which could be a limitation when extrapolating our findings to the clinical setting. Secondly, the treatment of postoperative ileus tends to be an ongoing process, while in our study; animals followed a strict protocol treatment, with no additional interventions. Finally, no histological analysis was performed in order to evaluate the presence of inflammatory cells within the muscular layers, which could add further information regarding the anti-inflammatory properties of the EA. Despite of these limitations, we were able to show that pre- and post-treatment with EA decreases the expression of proinflammatory cytokines in the intestinal wall and ameliorates intestinal dysmotility. Our findings support that the idea that EA might be a useful agent for attenuation and/or treatment of POI. Further studies are necessary to evaluate the efficacy and safety of the perioperative use of EA in the clinical setting.

## AUTHOR CONTRIBUTIONS

Harada T, Fink M and Cruz Jr RJ were responsible for the conception and design of the study, and analysis and interpretation of data. Harada T and Cruz Jr RJ were responsible for the data acquisition and approved the final version of the manuscript.
